# Breastfeeding grief after chest masculinisation mastectomy and detransition: A case report with lessons about unanticipated harm

**DOI:** 10.3389/fgwh.2023.1073053

**Published:** 2023-02-03

**Authors:** Karleen D. Gribble, Susan Bewley, Hannah G. Dahlen

**Affiliations:** ^1^ School of Nursing and Midwifery, Western Sydney University, Parramatta, NSW, Australia; ^2^ Department of Women and Children’s Health, King’s College London, London, United Kingdom

**Keywords:** breastfeeding, case report, detransition, grief, mastectomy, milk banking, transgender

## Abstract

An increasing number of young females are undergoing chest masculinsation mastectomy to affirm a gender identity and/or to relieve gender dysphoria. Some desist in their transgender identification and/or become reconciled with their sex, and then revert (or detransition). To the best of our knowledge, this report presents the first published case of a woman who had chest masculinisation surgery to affirm a gender identity as a trans man, but who later detransitioned, became pregnant and grieved her inability to breastfeed. She described a lack of understanding by maternity health providers of her experience and the importance she placed on breastfeeding. Subsequent poor maternity care contributed to her distress. The absence of breast function as a consideration in transgender surgical literature is highlighted. That breastfeeding is missing in counselling and consent guidelines for chest masculinisation mastectomy is also described as is the poor quality of existing research on detransition rates and benefit or otherwise of chest masculinising mastectomy. Recommendations are made for improving maternity care for detransitioned women^1^. Increasing numbers of chest masculinsation mastectomies will likely be followed by more new mothers without functioning breasts who will require honest, knowledgeable, and compassionate support.

## Introduction

Female individuals who experience a gender identity in conflict with their sex and/or who suffer from gender dysphoria may seek surgery to construct a male-appearing chest (1). This surgery is usually a type of subcutaneous mastectomy variously called “chest masculinisation”, “chest reconstruction”, “chest contouring”, or “top” surgery (2, 3). The surgical purpose is to affirm a gender identity as a trans man or non-binary person and/or to relieve psychological distress (1). Some breast tissue may be retained, unlike mastectomy for breast cancer, as aesthetic outcome is the priority (4).

Most transgender guidelines do not include the impact of chest masculinising mastectomy on breastfeeding as a part of the surgical consent process. Notably, the World Professional Association for Transgender Health (WPATH) Standards of Care makes no recommendation for counselling on breastfeeding before surgery (5) and nor do guidelines from Australia (AusPATH) (6) or New Zealand (PATHA) (7). Falck et al. (8) considered the experience of six transgender individuals who had chest masculinising surgery. They found the surgeon raised the impact on breastfeeding in just one case. This discussion occurred only because the patient had requested breast reduction (rather than chest masculinisation) and had not advised the surgeon of their transgender identification (8). This suggests a double standard may be at play in terms of warning patients about harms dependent on identity rather than procedure.

The impact of different surgical techniques for chest masculinisation on breastfeeding is absent from the literature. Research on ordinary breast reduction surgery shows that where the nipple, areola and breast tissue underneath the areola remain in place (so-called “pedicle” techniques) some milk making and milk removal capacity may be retained (9). However, when the nipple-areolar complex is separated from underlying glandular tissue, milk removal is impossible (9). The most common chest masculinisation technique involves separation of the nipple-areola complex from underlying tissue and excision of the nipple and areola which are then grafted back onto the reduced breasts in what is called “free nipple grafting” (10). Nipple reduction is a common adjunct, for which variety of techniques are used (4, 11); many result in a modified nipple with no functional orifices for milk removal [e.g., (12)].

It has been falsely claimed it is not possible to predict breastfeeding outcomes after chest masculinisation surgery based on surgical technique (13). Where surgery removes and grafts the nipple-areola complex, there is little to no possibility of milk removal from the nipple, even should glandular tissue remain. Where the nipple is kept in place but tissue underneath it removed and duct connections cut or nipple integrity forfeited, milk removal is also impossible. Furthermore, surgical complications such as necrosis can result in nipple loss (4, 14, 15) and surgery that removes the nipple and areola entirely may be chosen (16, 17). Considered together, these factors mean that many, if not most, individuals who have undergone chest masculinisation mastectomy, are unlikely to retain ability to both produce and extract milk. Proper discussion is required for the patient to choose and consent. Without recognising that the future will include pregnancy for at least some patients, surgeons cannot offer a conservative approach; either of deferring surgery or attempting to preserve some function.

The only breastfeeding-focussed research including participants who underwent chest masculinisation surgery is unfortunately unclear on the lactation and breastfeeding outcomes of all study participants (1). However, two individuals produced some milk that exited *via* their nipples; it seems in these cases, their surgeries did not involve nipple grafts and it can be assumed that some underlying breast tissue was retained. A further case involved an individual who had nipple grafts, sought to breastfeed but was unable to produce milk (1).

Some people do not persist in a transgender identification, and/or become reconciled with their sex, and detransition (18). Social detransition may involve presenting in a way more typical for their sex, reverting a name change, using sex-based pronouns, or overtly rejecting a transgender identification (19). Medical detransition usually involves stopping cross-sex hormones and require sex hormone replacement therapy in cases of gonadectomy (19). The experiences of detransitioners have been little studied, but transition regret is commonly reported in existing research (18, 19). Young and childless detransitioners who had mastectomies have spoken specifically of regret about inability to breastfeed (20, 21).

Case reports are a timely way for increasing knowledge of unusual or new conditions or circumstances and so help inform healthcare (22). They place the “*care and treatment of the individual patient centre-stage”* (23), can be valuable as an “*early warning signal”* and contribute to the health and wellbeing of others in the future (24). This paper presents a case report of a woman who identified as transgender and obtained a chest masculinisation mastectomy but later detransitioned. She experienced intense grief around her inability to breastfeed her infant. During a three-hour interview with the first author (KG), the woman, whom we are calling Elizabeth, told her story of transition, detransition, pregnancy, birth, and new motherhood. She also provided the authors with documentary support for her account including pregnancy medical records, her referral to the milk bank which described her reasons for seeking banked donor milk, and photographs of her mastectomy scarring. KG with the assistance of the second author (SB) developed the case description based on the transcribed interview in consultation with Elizabeth, with a focus on her experiences and feelings regarding her breasts, mastectomy, and breastfeeding and the impact of this on her as a pregnant woman and new mother. Some details have been changed to preserve anonymity. Written consent for publication and approval of the finalised paper was obtained from Elizabeth. Ethical approval for publication was granted by the Human Research Ethics Committee of Western Sydney University (approval H14913). The detailed experience of detransitioned women who had chest masculinisation mastectomies and then became mothers has not, to our knowledge, previously been described. This case report provides guidance to assist health professionals to better support detransitioned women who become mothers.

## Case description

Elizabeth is a detransitioned woman in her thirties. She first experienced discomfort in her female body as a 10-year-old when she developed breasts. She described being teased by other children and “*getting sexually harassed by adult men”* as *“really negative experiences”* that led her to hate her breasts. At age 15 years, Elizabeth heard about the concept of gender identity and became persuaded that her bodily discomfort was because she was transgender. She started wearing a sports bra to flatten her breasts, represented herself as a boy, and changed her name legally. At age 18, Elizabeth sought treatment at an adult gender identity service, was formally diagnosed with gender identity disorder, and was prescribed testosterone. At age 19, she obtained referral for chest masculinisation mastectomy and at 20 years she had a double incision subcutaneous mastectomy with free nipple grafts (25). She recollects no discussion of the impact on breastfeeding in this process.

The surgical outcome was not what was expected. There was extensive hypertrophic scarring, particularly around and under the nipple grafts, which were painful and itchy to the extent that wearing clothing was uncomfortable. Elizabeth describes her right nipple graft chronically leaking a watery fluid, while voids in her scarred left nipple graft accumulated a smelly paste that had to be regularly squeezed out. Two years later, she underwent surgery to reduce scars; leaving her chest sunken and nipple-less while scarring and nerve pain remained. Elizabeth felt unsupported by her transgender friends; they saw her surgical outcome as reflecting badly on transition and told her not to tell others about her experience because, “*you’re making trans surgery look bad.”* She describes how she, “*went from being a trans activist… to being persona non grata because I was complaining about these botched chest surgeries, and it was just really devastating.”* The failure of the surgery to fulfil its promise, concurrent with rejection by transgender friends and testosterone-induced vaginal atrophy, resulted in a psychological crisis. Elizabeth abandoned gender identity as a useful framework for understanding herself and the idea that she was a man of any kind. At age 24, she detransitioned, stopping testosterone, disassociating from the transgender community, and considering her previous identification and medicalisation a “*terrible mistake”*.

Although her surgeon did not discuss breastfeeding, Elizabeth believed that if he had she would not have welcomed the conversation, “*I don't think I would have been receptive, I would have felt insulted and I would have said it was triggering my gender dysphoria.”* However, Elizabeth explains that this response would have been an avoidance tactic, “*That wouldn't really have been true. It would have been because…maybe I did want children… but it's like this trump card, gender dysphoria, meaning you can't have any conversation.”* That is, mention of gender dysphoria stops health professionals from further exploration. She explains, *“I was conflicted about* [possible future motherhood]*… because part of me actually did want children. I was very confused about, could I be an OK mother? Or would I be a bad mother? And so to me, rather than having to deal with those questions, the easiest thing was to make it impossible… take the choice away.”*

Over subsequent years, Elizabeth determined she really did want children and then worried about whether she would be fertile because of prior testosterone use. She was also concerned about her inability to breastfeed, “*It was clear to me that I wanted to give my child the best possible start in life. And I did all the research, I knew that breastfeeding was really important.”* She contemplated not having a child because she couldn’t breastfeed. However, at age 30, after a year of trying to conceive, Elizabeth became pregnant. The pregnancy was complicated by gestational diabetes but otherwise unremarkable. During one antenatal appointment with her midwife, Elizabeth expressed a desire to obtain donor milk. Her midwife reacted negatively and dismissively, saying she should “*just formula feed.”* In seeking understanding, Elizabeth shared her distress at not being able to breastfeed, her concern that she would be an inadequate mother because of this and described the trauma of her breast surgeries. Elizabeth's assessment is that her midwife was shocked; it was clear she had no experience of women without breasts following chest masculinisation mastectomy. The midwife also did not understand Elizabeth's anguish about her breastfeeding inability which only increased her distress such that a referral was made to an obstetrician.

Seemingly, this obstetrician had received training on the care of transgender people but he did not understand Elizabeth had detransitioned. She describes how, “*[the] doctor decided he was going to straighten things out, because he thought that, well, ‘this old midwife just doesn't understand that this is a trans man and I'm going to fix things by correctly gendering this birthing parent, and then, you know, he'll calm down and everything will be great.’* He persistently referred to Elizabeth as ‘a man’ which Elizabeth found confusing and frightening. She tried to explain that she was not transgender and was not male. She wondered whether the obstetrician thought she was a mentally ill trans woman who mistakenly believed she was pregnant and was just being humoured. Alternatively, she considered whether the doctor was incompetent, persuaded by ideology that it was possible for a male to become pregnant. Either possibility made Elizabeth fear she would receive poor maternity care. Elizabeth's distress and her health providers” confusion resulted in a child protection report. It was only with hindsight that Elizabeth realised that the doctor must have been trained to “affirm” transgender identification and to prioritise gender identity over sex.

Elizabeth's infant was placed skin-to-skin on her chest after a caesarean birth and he sought her breast. She describes how, “*it was really hard knowing that he wanted to breastfeed, and I couldn't give him that…And when, when they put him on my stomach, he crawled up, he was looking for my breasts, and he couldn't find them. And he tried to suck on my chin. And he spent so much time in his early life trying to find my breasts.”*

Elizabeth was successful in obtaining banked donor milk; the milk bank manager was sympathetic to her circumstances, and thanks to Elizabeth's gestational diabetes, her infant met the eligibility criteria. While grateful for the respect given at the milk bank and for the milk itself, and despite knowledge that sick infants were prioritised in the system, Elizabeth felt guilty about taking this milk. She said, *“I always had the feeling that even if I knew that the babies who really needed it more, were getting served first, I was worried that a baby who might have needed it was going to be deprived because of me.”* Elizabeth also obtained breastmilk directly from other mothers *via* social media. The combination of banked donor milk and peer-to-peer shared milk meant her infant was exclusively breastmilk fed for two months and mixed fed breastmilk and infant formula for a further two months.

Elizabeth had hoped that providing her son with donor breastmilk would alleviate her guilt and grief. However, this didn't occur. Seeing her infant exhibiting feeding cues and having to get up to make a bottle, instead of offering the breast, was difficult, “*I'd be cuddling with him. And I could feel like this is the time he would want the breast. It was so obvious.”* She recognised a relational aspect to breastfeeding they both missed.

Elizabeth's adjustment to new motherhood was made more stressful due to the child protection report follow up. Shortly after birth she had to, “*see a social worker from the child protective services and then go to a psychiatrist to get a mental health evaluation to prove that I wasn't insane…it was honestly traumatising.”* While no mental health concerns were identified and her case was closed, it nevertheless impacted her negatively. She said, “*We got off to a kind of a difficult start because of the stress of that…I just felt very misunderstood and very vulnerable, and very judged and inadequate.”*

Seeing her infant search for her breast was challenging, but Elizabeth looks back at the time spent skin-to-skin with fondness and gratitude. She says, “*I didn’t avoid it because of* [baby hunting for the breast]… *I'm really glad that that we did cuddle a lot when he was a baby… I'm glad that I spent a lot of time with him on my chest even though I couldn't actually breastfeed*.” Elizabeth says she now has a good relationship with her son who calls her “mummy” and that both these things helped her greatly. She is often misidentified as a trans woman due to her deep voice and flat chest but this does not bother her as it used to. Learning about other detransitioners post-pregnancy helped her, “*to have some closure, where, you know, I don't feel alone with the stuff anymore and I now have vocabulary so I can describe my experiences.”*

Elizabeth wants to raise awareness of the experiences of others like her, “*to help try to make things less uncomfortable for pregnant, or shortly after pregnancy, detransitioned women who are trying to feed their babies.”* She wants health providers to know that, “*you can't assume based on how someone looks that they believe in gender identity and that they are going to want to be interacted with as if they are transgender*.” She wants people to be aware of potential complications associated with lactation following chest masculinisation mastectomy: for example, some will have remaining breast tissue but no working nipple or connection to the nipple and may develop engorgement; others may seek to feed their infant at their breast using a breastfeeding supplementer. However, this may be prevented or complicated by lack of breast tissue for the infant to latch onto or nipple grafts that are at high risk of damage due to compromised blood circulation and poor or absent sensation. She notes that those who retain a transgender identity may be reluctant to speak about their challenges because of social pressure to put a positive face on transition. She also suggests that for some their distress may be expressed as gender dysphoria rather than as breastfeeding grief, “*gender dysphoria is something that can conceal more than it reveals and somebody who's distressed because they realise they can't breastfeed their baby, that distress* [may be] *interpreted and expressed as gender dysphoria, rather than the actual cause.”* Such mothers (or fathers as they may wish to be called) and infants, she says, will need sensitive support.

Elizabeth is keenly aware of how lucky she was to be able to have a baby. This, to some extent, was a saving grace. She describes how she felt when trying to get pregnant, “*I had this feeling, if I'm able to still become a mother, then this whole transition, and how that was a miserable failure and mistake will just be a footnote in my life… but if I'm not able to have a child, then I will spend the rest of my life and go to my grave, having this combined regret of the transition and the childlessness together.”* She remains concerned about the potential impact of prior testosterone, “*I'll never be able to forgive myself for whatever potential effects of testosterone that I took might have had on my eggs and affected my son including, epigenetic changes… who knows what that did?”*

Because of her own experience, Elizabeth is highly critical of the quality of medical care provided to people with gender dysphoria, “*I'm really sad that there's going to be so many women, many of whom are children today, who are not going to be able to have children of their own because they're being sterilised. And I'm very frustrated, that it's taboo to talk about that… I am speaking because I want to spare future mothers and babies what we went through if I can.”*

## Discussion

This case report exposes issues related to the undervaluing of breastfeeding in transgender medicine and maternity care. Transgender guidance routinely makes no mention of a need to discuss the impact of chest masculinisation surgery on breastfeeding, so it is perhaps unsurprising that Elizabeth's surgeon seemingly did not discuss this. Literature on the pros and cons of surgical techniques for chest masculinisation do not discuss the impact on breastfeeding [e.g., (2, 26, 27)]. Several authors have stated—wrongly—that those who regret surgery can have a “reversal” of their mastectomy (28, 29), apparently failing to appreciate that mastectomy involves removal of an organ and permanent loss of function.

The midwife was dismissive and confused by Elizabeth's distress and did not understand why she sought donor breastmilk. However, an abundance of research shows that Elizabeth was not unusual in seeing breastfeeding as consequential [e.g., (30, 31–34)]. Her reasons for placing importance on breastfeeding align with those previously identified: breastfeeding is a biologically normal, instinctive behaviour, intrinsically linked with motherhood, is a way of being in relationship with one's infant, and is important to infant health (35). Women who want, but are unable, to breastfeed are at increased risk of postnatal depression (36, 37). Dismissing their breastfeeding grief merely increases distress and decreases trust in health providers.

The other major source of distress for Elizabeth was lack of understanding of her situation as a detransitioner. Her midwife appeared unaware of gender transition and her obstetrician was unaware of detransition. Elizabeth's fear that her obstetrician believed she was a pregnant male might seem far-fetched, but there are examples of individuals and organisations becoming confused about or believing impossible biology. Recently, student midwives at one university were provided with teaching materials on how to catheterise someone with a penis and told they may be required to care, “*for a pregnant or birthing person who is transitioning from male to female and may still have external male genitalia”* (38). This particular error (which was quickly corrected) seems likely related to the increasingly common use of language suggesting that a person of either sex may give birth or overt claims that men may become pregnant (39). Incongruence between pregnancy as an exclusively female experience and language stating otherwise may result in mistakes, similar to the “Stroop Effect” where conflicting stimuli introduce errors (40).

Other examples of erroneous health care advice exist. For example, the Canadian Cancer Society indicates that trans women may need cervical cancer screening (41). The potential for incongruent language and ideological infiltration to cause confusion, compromise care, and undermine patient confidence needs to be better appreciated. Certainly, maternity care providers need to be aware that detransitioners exist; they may have medical needs related to previous hormone treatment or surgery and they may overtly reject the concept of gender identity. Continuity of midwifery care should reduce the chance that the care itself becomes a source of psychological distress for detransitioned women. Continuity of care is shown to optimise physical and psychological outcomes (42, 43) and is highly valued by women (44). A key advantage is that women can share their story once with the lead care provider who then advocates for them across the childbearing continuum.

The responses of Elizabeth's midwife and obstetrician contrast with the individualised care at the milk bank. Research has demonstrated the psychological benefit of milk bank staff listening to women and validating their desire to provide their infants with breastmilk (45). However, Elizabeth's feelings of guilt are consistent with research showing that women who access banked donor milk, may feel concerned they are taking milk from other needy infants (45). This highlights the importance of emotional support for all mothers seeking donor milk.

Elizabeth's experience also highlights the need for better health support for detransitioners. What limited research exists, indicates detransitioners often have low trust in health providers (19, 46) and do not believe they were properly evaluated nor properly informed about treatment health implications or risks (18, 19). During detransition, many report finding health professionals lack knowledge or are dismissive of feelings of being harmed by transition and of their health care needs (19). However, detransitioners recognise they need health care, including psychological support, with the majority wanting assistance coping with transition regret (19). Assistance may be most critical during the acute detransition, but may also be needed when transition consequences are experienced, such as if facing fertility issues or being unable to breastfeed. As Elizabeth found, peer-support from other detransitioners is valuable (46). It is noteworthy that over a third of female detransitioners in existing research had chest masculinising mastectomy (18, 19).

The information deficit and lack of consideration about breastfeeding for those contemplating chest masculinisation mastectomy must be addressed. Guidelines such as those from WPATH, AusPATH and PATHA should require discussion about the impact of chest masculinising surgery on breastfeeding; it is surely unethical (and arguably negligent) for surgeons to remove an organ without explaining the functional effect to patients. Furthermore, guidance for clinicians supporting new mothers needs to be clearer on the likely impact of chest masculinisation surgery and potential breastfeeding complications. Current publications skirt around the issue and are not clear that the most common chest masculinisation surgical technique almost certainly precludes breastfeeding in terms of providing milk to an infant. They do not mention foreseeable complications such as engorgement from remaining breast tissue with no patent nipple orifices or that poor blood flow and enervation to nipple grafts increase risk of nipple damage and infections (1, 47–51) and may make it inadvisable for an infant to suckle. Current guidance also neglects emotional support for breastfeeding inability while even those who retain their transgender identification may find their inability to breastfeed difficult. As an example, the prominent UK trans man Freddie McConnell expressed sadness about being unable to provide any milk for his infant and described wishing his surgeon had maintained nipple placement so that the possibility of “chestfeeding” was retained (52). As Butler and Hutchinson (53) describe, there is a “*pressing need for research and services for gender desisters/detransitioners”.* Support must include assistance for women who are unable to breastfeed but also those who are infertile—either because they never fully developed due to puberty blocking medication, or because they used testosterone or underwent hysterectomy.

There is widespread concern about the rise in numbers of children attending gender clinics: 13-, 14-, and 19-fold increases in Norway, the United Kingdom, and Sweden respectively 2011–2017 (54). The proportion of girls attending UK gender clinics rose from 50% to 75% female 2011–2019 (20). In many countries, chest masculinisation mastectomies are carried out on minors, with reports of surgical referral for 12 year olds (28) and surgery undertaken on 13 year olds (3). One clinic in the USA reported a 13-fold increase in minor females referred for mastectomy 2013–2020 (28). Discussion of future inability to breastfeed is also absent in publications relating to minors [e.g., (28, 55, 56)]. One authority was recorded saying that adolescents who “*want breasts at a later point… can go and get them”* (56) demonstrating startling ignorance of breast function. A small retrospective study of 14 adolescents (1 month to 3.6 year follow up), found one young adult stopped testosterone two months after chest masculinisation surgery and asked to revert her sex marker to female five months later (57). Another retrospective study with 68 adolescents and young adults (13–24 years at surgery and one to five year follow up) found one individual reported surgical regret “sometimes” (3).

Detransition and regret about hormone administration or transition surgery are frequently said to be rare or exceedingly rare with low detransition rates <3% (5, 58–61). The research upon which such claims are made suffers from a number of serious limitations: short follow-up [e.g., (62)]; high [e.g., (63)] or unclear rates of loss to follow up [e.g., (4)]; reliance on individuals returning to secondary care clinics reporting transition regret or seeking reversal procedures [e.g., (64)]; reliance on reversion of legal sex absent knowledge of the experiences or views of those who have not sought legal reversion [e.g., (60, 65)]; errors and non-replicability (4, 66–69). Research suggesting much higher rates of detransition [7%–30% (70, 71)] also has flaws, meaning that detransition rates may be under- or over- reported. Short follow-up is particularly concerning as it is evident that transition regret may have long latency; in one large cohort study the average time to regret was 10 years (63). Thus, there has not been time for long-term follow up of the newer cohort of younger women who have transitioned in the past decade.

Research specifically regarding chest masculinsation mastectomy for minors and young adults, is of poor quality and makes surprisingly confident statements of benefit. For example, Ascha et al. (72), considered the psychological impact of chest masculinisation mastectomy on 14–24 year olds but had only a three month follow up and 14% drop out. They argued that there is no evidence to support delaying chest masculinisation mastectomy based on age and claimed that their findings would, “*help dispel misconceptions that gender-affirming treatment is experimental.”* This paper, published in the highly ranked journal JAMA Pediatrics, was accompanied by an editorial entitled, “*Top surgery in adolescents and young adult- effective and medically necessary”* (61). Such claims cannot be substantiated and call into question whether there has been a failure of peer review and the editorial process. Children and young adults with gender dysphoria deserve a good research basis for their treatment and a critical consideration of the risks and benefits of procedures like chest masculinisation mastectomy (73). The number of people currently undergoing chest masculinisation mastectomy is increasing (74). Given that many women have their first baby in their 30s, there may be a decade or two between surgery and pregnancy during which regret may surface. In the future, there may be many mothers without breasts, needing support around inability to breastfeed.

## Strengths and limitations

Strengths of this case study include the rich detail Elizabeth could provide about her own experience of giving birth and feeding her infant and her perspective of the attending health professionals. Limitations are that it is unknown how representative this is of detransitioned women.

## Recommendations for practice

• Health systems and organisations, including breastfeeding organisations, should advocate with gender identity services, surgical associations, and transgender guideline producers to appreciate the importance of breast function and breastfeeding. They should ensure their own publications about chest masculinisation mastectomy provide honest information about the barriers surgery poses to breastfeeding and the potential complications. Quality research on the long-term benefits or otherwise of chest masculinisation mastectomy, particularly regarding adolescents, is urgently needed.
• Gender identity clinicians must provide patients with more information before undertaking chest masculinisation surgery, given that the future is not predictable and because they will otherwise be open to medico-legal challenges. This must include implications for infant feeding, even if pregnancy is not immediately contemplated.
• Awareness needs to be raised about the existence of detransitioned women who may require maternity care. Sensitive, informative content on detransitioners should be embedded in undergraduate midwifery and medical curricula. Assumptions should not be made about the gender identity of any individual based on their appearance, or indeed whether individuals have a gender identity.
• Continuity of midwifery care should be provided as standard and personal preferences regarding how individuals are referred to should be adhered to. Alongside sex (75), hospital and practice intake forms should collect data on gender identity, including an option of “I do not have a gender identity” or “not applicable.”
• Antenatally, women who have had chest masculinisation mastectomy should be provided with assistance to consider their options for infant feeding. This should include discussion of the type of surgery and the condition of their nipples and areolae. They should also be informed on what to expect postnatally concerning engorgement or likelihood of lack of milk production or extraction.
• Pregnant women and new mothers who have had chest masculinisation surgery should be advised about the importance of skin-to-skin for infants, especially if they are unable to breastfeed. Anticipatory guidance about their infant's instinctive breast-seeking behaviour needs to be provided with emotional support available for any distress. Support around responsive bottle feeding should also be provided where an infant is not breastfed.
• After birth, skilled care should be provided to women who have had chest masculinising mastectomy and who wish to initiate breastfeeding. Where attachment at the breast is possible, particular attention should be paid to optimising latch, preventing nipple damage and infections, and ensuring the infant is drinking sufficient milk at the breast or otherwise.
• All women who express guilt, sadness, or regret about being unable to breastfeed their infants, regardless of the reasons, should have their experience and feelings respected and be provided with appropriate emotional support. Health providers should be aware that transgender individuals may find it difficult to verbalise their distress because of a felt need to present transition interventions positively.
• Milk banks should consider eligibility for donor human milk for women who have had mastectomies on the basis of supporting the infant's right to health (76).

## Conclusions

Until recently, it would be extremely unusual for a new mother to have had a mastectomy. With increasing numbers of female adolescents and young adults obtaining chest masculinisation mastectomies, more new mothers will present without functioning breasts. Some will retain a transgender identification and others will have detransitioned. Each requires an individualised approach to provide them and their infants with honest, knowledgeable, and compassionate support.

## Data Availability

The original contributions presented in the study are included in the article, further inquiries can be directed to the corresponding author.
